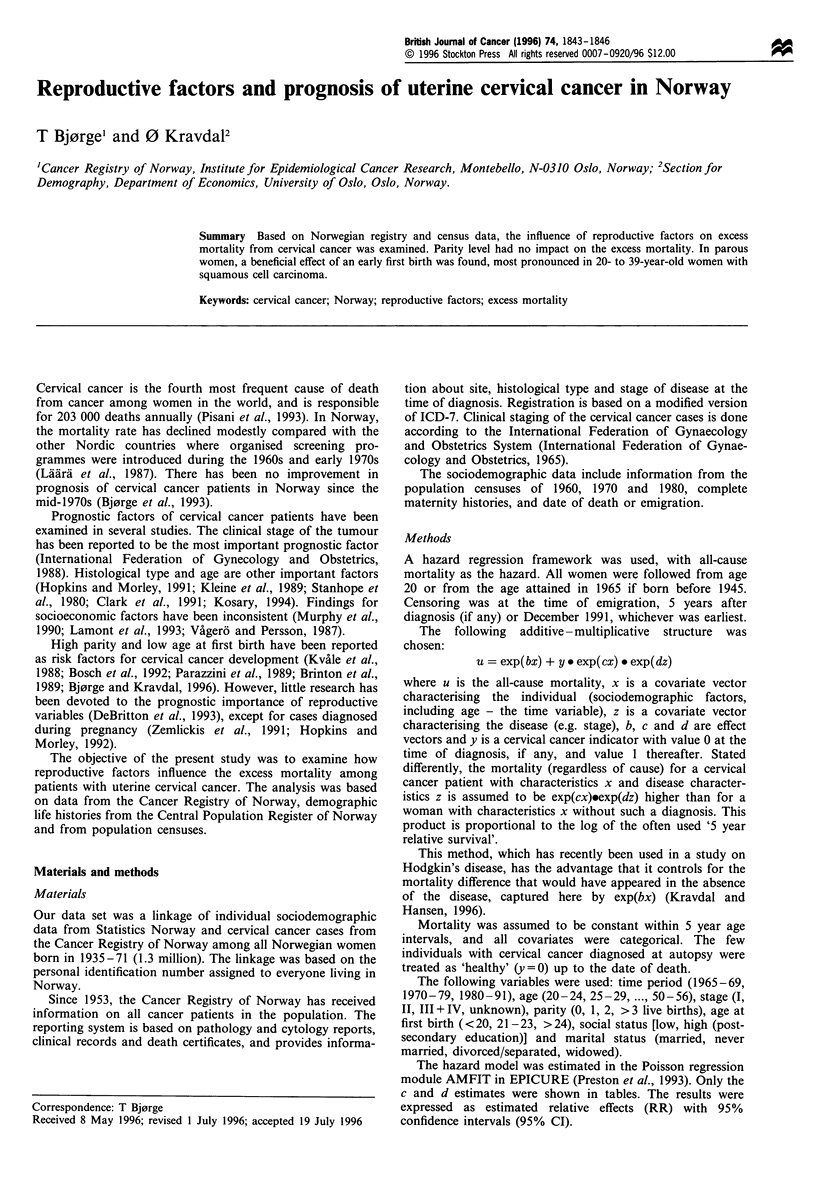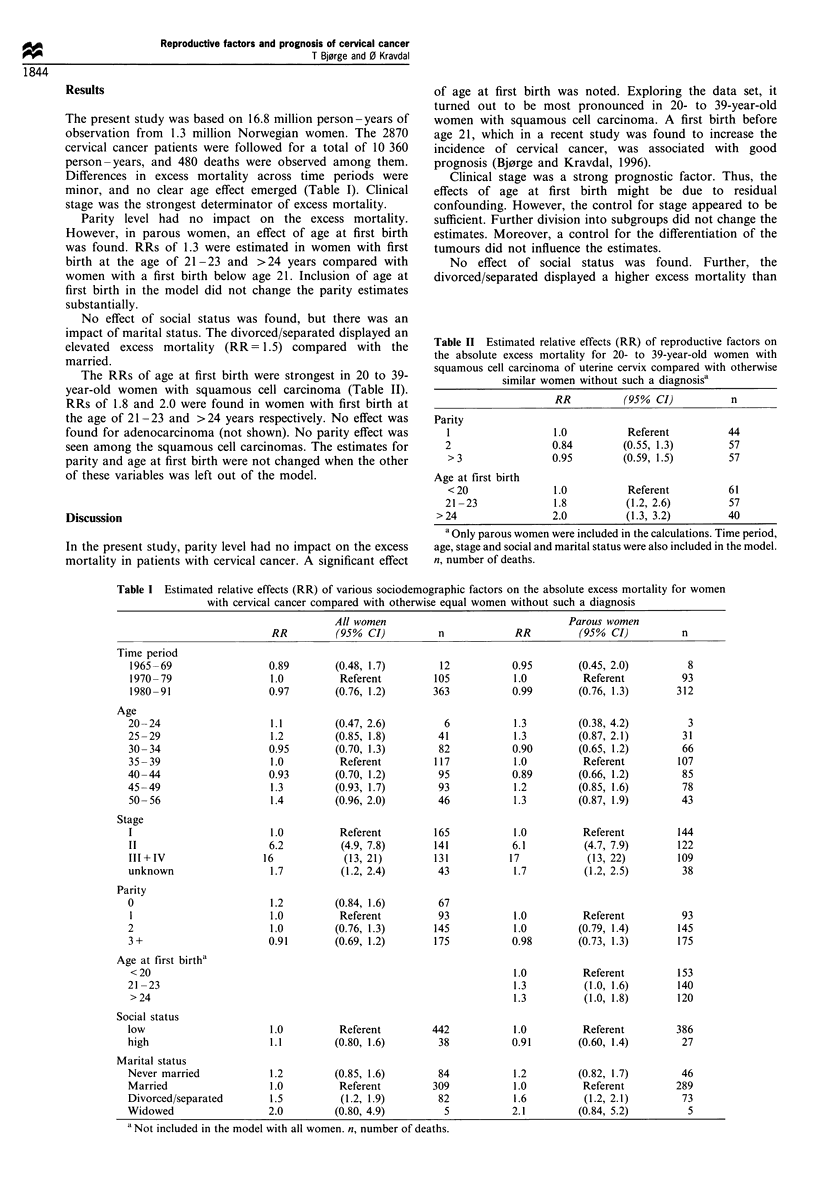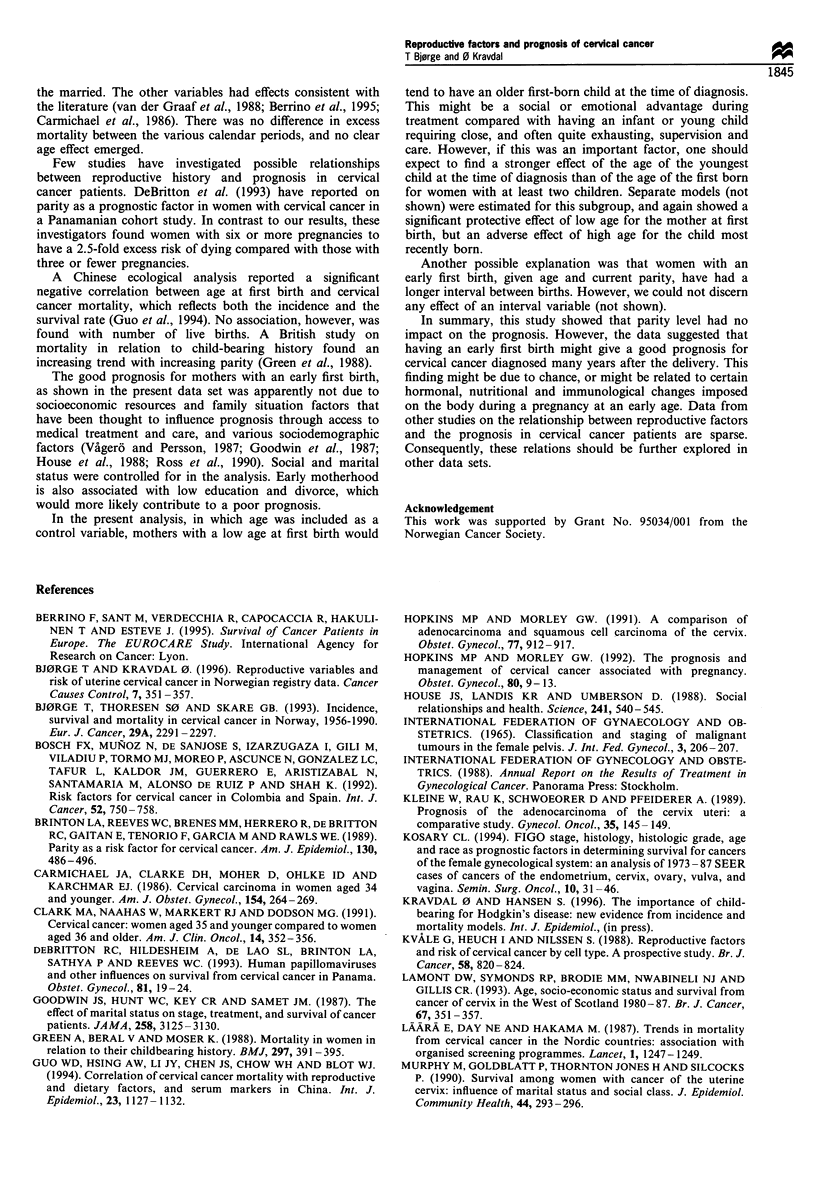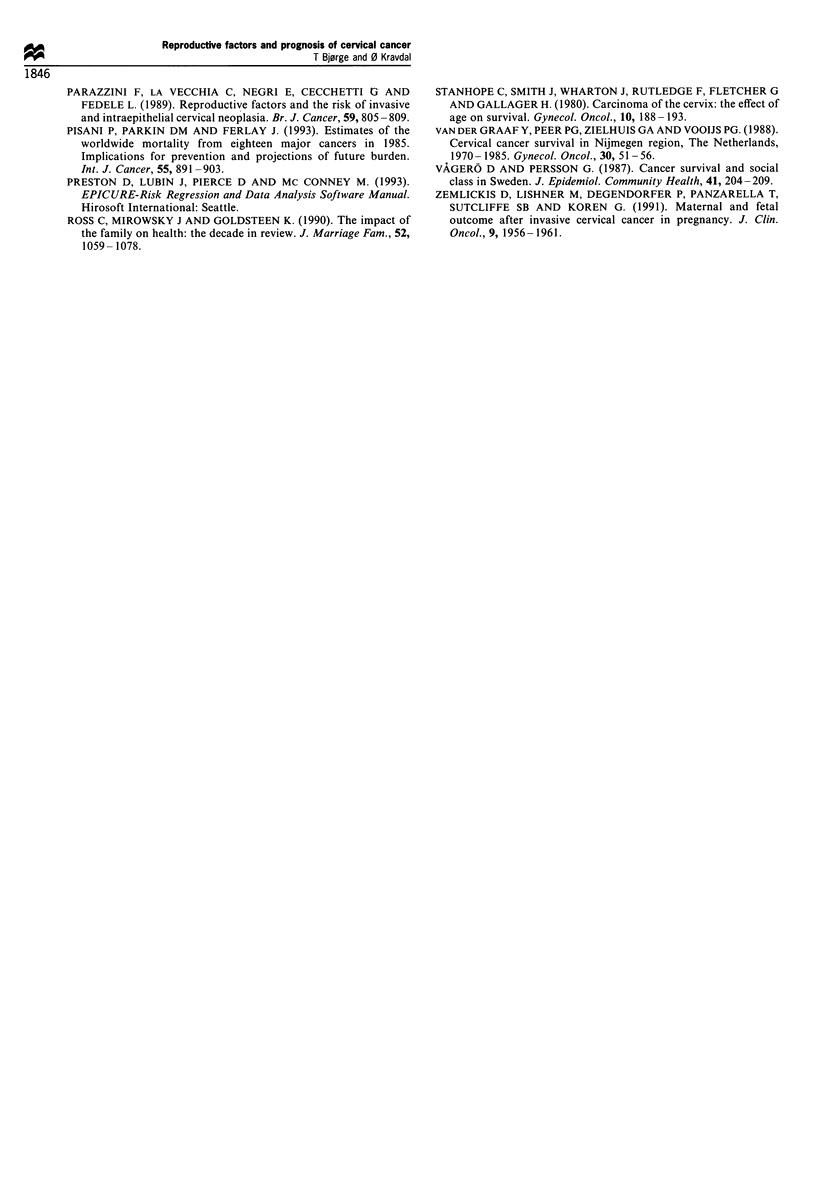# Reproductive factors and prognosis of uterine cervical cancer in Norway.

**DOI:** 10.1038/bjc.1996.641

**Published:** 1996-12

**Authors:** T. Bjørge, O. Kravdal

**Affiliations:** Cancer Registry of Norway, Institute for Epidemiological Cancer Research, Mantebello, Oslo, Norway.

## Abstract

Based on Norwegian registry and census data, the influence of reproductive factors on excess mortality from cervical cancer was examined. Parity level had no impact on the excess mortality. In parous women, a beneficial effect of an early first birth was found, most pronounced in 20- to 39-year-old women with squamous cell carcinoma.


British Journal of Cancer (1996) 74, 1843-1846
© 1996 Stockton Press All rights reserved 0007-0920/96 $12.00

### Reproductive factors and prognosis of uterine cervical cancer in Norway

T Bjørge[1] and Ø Kravdal[2]

[1]*Cancer Registry of Norway, Institute for Epidemiological Cancer Research, Montebello, N-0310 Oslo, Norway;* [2]*Section for Demography, Department of Economics, University of Oslo, Oslo, Norway.*

**Summary** Based on Norwegian registry and census data, the influence of reproductive factors on excess mortality from cervical cancer was examined. Parity level had no impact on the excess mortality. In parous women, a beneficial effect of an early first birth was found, most pronounced in 20- to 39-year-old women with squamous cell carcinoma.

**Keywords:** cervical cancer; Norway; reproductive factors; excess mortality

Cervical cancer is the fourth most frequent cause of death from cancer among women in the world, and is responsible for 203 000 deaths annually (Pisani *et al.*, 1993). In Norway, the mortality rate has declined modestly compared with the other Nordic countries where organised screening programmes were introduced during the 1960s and early 1970s (Läärä *et al.*, 1987). There has been no improvement in prognosis of cervical cancer patients in Norway since the mid-1970s (Bjørge *et al.*, 1993).

Prognostic factors of cervical cancer patients have been examined in several studies. The clinical stage of the tumour has been reported to be the most important prognostic factor (International Federation of Gynecology and Obstetrics, 1988). Histological type and age are other important factors (Hopkins and Morley, 1991; Kleine *et al.*, 1989; Stanhope *et al.*, 1980; Clark *et al.*, 1991; Kosary, 1994). Findings for socioeconomic factors have been inconsistent (Murphy *et al.*, 1990; Lamont *et al.*, 1993; Vågerö and Persson, 1987).

High parity and low age at first birth have been reported as risk factors for cervical cancer development (Kvåle *et al.*, 1988; Bosch *et al.*, 1992; Parazzini *et al.*, 1989; Brinton *et al.*, 1989; Bjørge and Kravdal, 1996). However, little research has been devoted to the prognostic importance of reproductive variables (DeBritton *et al.*, 1993), except for cases diagnosed during pregnancy (Zemlickis *et al.*, 1991; Hopkins and Morley, 1992).

The objective of the present study was to examine how reproductive factors influence the excess mortality among patients with uterine cervical cancer. The analysis was based on data from the Cancer Registry of Norway, demographic life histories from the Central Population Register of Norway and from population censuses.

### Materials and methods

### *Materials*

Our data set was a linkage of individual sociodemographic data from Statistics Norway and cervical cancer cases from the Cancer Registry of Norway among all Norwegian women born in 1935–71 (1.3 million). The linkage was based on the personal identification number assigned to everyone living in Norway.

Since 1953, the Cancer Registry of Norway has received information on all cancer patients in the population. The reporting system is based on pathology and cytology reports, clinical records and death certificates, and provides information about site, histological type and stage of disease at the time of diagnosis. Registration is based on a modified version of ICD-7. Clinical staging of the cervical cancer cases is done according to the International Federation of Gynaecology and Obstetrics System (International Federation of Gynaecology and Obstetrics, 1965).

The sociodemographic data include information from the population censuses of 1960, 1970 and 1980, complete maternity histories, and date of death or emigration.

### *Methods*

A hazard regression framework was used, with all-cause mortality as the hazard. All women were followed from age 20 or from the age attained in 1965 if born before 1945. Censoring was at the time of emigration, 5 years after diagnosis (if any) or December 1991, whichever was earliest.

The following additive–multiplicative structure was chosen:

$$u = \exp(bx) + y \bullet \exp(cx) \bullet \exp(dz)$$

where $u$ is the all-cause mortality, $x$ is a covariate vector characterising the individual (sociodemographic factors, including age – the time variable), $z$ is a covariate vector characterising the disease (e.g. stage), $b$, $c$ and $d$ are effect vectors and $y$ is a cervical cancer indicator with value 0 at the time of diagnosis, if any, and value 1 thereafter. Stated differently, the mortality (regardless of cause) for a cervical cancer patient with characteristics $x$ and disease characteristics $z$ is assumed to be $\exp(cx) \bullet \exp(dz)$ higher than for a woman with characteristics $x$ without such a diagnosis. This product is proportional to the log of the often used '5 year relative survival'.

This method, which has recently been used in a study on Hodgkin's disease, has the advantage that it controls for the mortality difference that would have appeared in the absence of the disease, captured here by $\exp(bx)$ (Kravdal and Hansen, 1996).

Mortality was assumed to be constant within 5 year age intervals, and all covariates were categorical. The few individuals with cervical cancer diagnosed at autopsy were treated as 'healthy' ($y=0$) up to the date of death.

The following variables were used: time period (1965–69, 1970–79, 1980–91), age (20–24, 25–29, ..., 50–56), stage (I, II, III+IV, unknown), parity (0, 1, 2, >3 live births), age at first birth (<20, 21–23, >24), social status [low, high (post-secondary education)] and marital status (married, never married, divorced/separated, widowed).

The hazard model was estimated in the Poisson regression module AMFIT in EPICURE (Preston *et al.*, 1993). Only the $c$ and $d$ estimates were shown in tables. The results were expressed as estimated relative effects (RR) with 95% confidence intervals (95% CI).

Correspondence: T Bjørge
Received 8 May 1996; revised 1 July 1996; accepted 19 July 1996

### Results

The present study was based on 16.8 million person-years of observation from 1.3 million Norwegian women. The 2870 cervical cancer patients were followed for a total of 10 360 person-years, and 480 deaths were observed among them. Differences in excess mortality across time periods were minor, and no clear age effect emerged (Table I). Clinical stage was the strongest determinator of excess mortality.

Parity level had no impact on the excess mortality. However, in parous women, an effect of age at first birth was found. RRs of 1.3 were estimated in women with first birth at the age of 21-23 and >24 years compared with women with a first birth below age 21. Inclusion of age at first birth in the model did not change the parity estimates substantially.

No effect of social status was found, but there was an impact of marital status. The divorced/separated displayed an elevated excess mortality (RR = 1.5) compared with the married.

The RRs of age at first birth were strongest in 20 to 39-year-old women with squamous cell carcinoma (Table II). RRs of 1.8 and 2.0 were found in women with first birth at the age of 21-23 and >24 years respectively. No effect was found for adenocarcinoma (not shown). No parity effect was seen among the squamous cell carcinomas. The estimates for parity and age at first birth were not changed when the other of these variables was left out of the model.

### Discussion

In the present study, parity level had no impact on the excess mortality in patients with cervical cancer. A significant effect of age at first birth was noted. Exploring the data set, it turned out to be most pronounced in 20- to 39-year-old women with squamous cell carcinoma. A first birth before age 21, which in a recent study was found to increase the incidence of cervical cancer, was associated with good prognosis (Bjørge and Kravdal, 1996).

Clinical stage was a strong prognostic factor. Thus, the effects of age at first birth might be due to residual confounding. However, the control for stage appeared to be sufficient. Further division into subgroups did not change the estimates. Moreover, a control for the differentiation of the tumours did not influence the estimates.

No effect of social status was found. Further, the divorced/separated displayed a higher excess mortality than

**Table II** Estimated relative effects (RR) of reproductive factors on the absolute excess mortality for 20- to 39-year-old women with squamous cell carcinoma of uterine cervix compared with otherwise similar women without such a diagnosis[a]

| | *RR* | *(95% CI)* | n |
|---|---|---|---|
| Parity | | | |
| 1 | 1.0 | Referent | 44 |
| 2 | 0.84 | (0.55, 1.3) | 57 |
| >3 | 0.95 | (0.59, 1.5) | 57 |
| Age at first birth | | | |
| <20 | 1.0 | Referent | 61 |
| 21-23 | 1.8 | (1.2, 2.6) | 57 |
| >24 | 2.0 | (1.3, 3.2) | 40 |

[a] Only parous women were included in the calculations. Time period, age, stage and social and marital status were also included in the model. *n*, number of deaths.

**Table I** Estimated relative effects (RR) of various sociodemographic factors on the absolute excess mortality for women with cervical cancer compared with otherwise equal women without such a diagnosis

| | *All women* | | | *Parous women* | | |
|---|---|---|---|---|---|---|
| | *RR* | *(95% CI)* | n | *RR* | *(95% CI)* | n |
| Time period | | | | | | |
| 1965-69 | 0.89 | (0.48, 1.7) | 12 | 0.95 | (0.45, 2.0) | 8 |
| 1970-79 | 1.0 | Referent | 105 | 1.0 | Referent | 93 |
| 1980-91 | 0.97 | (0.76, 1.2) | 363 | 0.99 | (0.76, 1.3) | 312 |
| Age | | | | | | |
| 20-24 | 1.1 | (0.47, 2.6) | 6 | 1.3 | (0.38, 4.2) | 3 |
| 25-29 | 1.2 | (0.85, 1.8) | 41 | 1.3 | (0.87, 2.1) | 31 |
| 30-34 | 0.95 | (0.70, 1.3) | 82 | 0.90 | (0.65, 1.2) | 66 |
| 35-39 | 1.0 | Referent | 117 | 1.0 | Referent | 107 |
| 40-44 | 0.93 | (0.70, 1.2) | 95 | 0.89 | (0.66, 1.2) | 85 |
| 45-49 | 1.3 | (0.93, 1.7) | 93 | 1.2 | (0.85, 1.6) | 78 |
| 50-56 | 1.4 | (0.96, 2.0) | 46 | 1.3 | (0.87, 1.9) | 43 |
| Stage | | | | | | |
| I | 1.0 | Referent | 165 | 1.0 | Referent | 144 |
| II | 6.2 | (4.9, 7.8) | 141 | 6.1 | (4.7, 7.9) | 122 |
| III+IV | 16 | (13, 21) | 131 | 17 | (13, 22) | 109 |
| unknown | 1.7 | (1.2, 2.4) | 43 | 1.7 | (1.2, 2.5) | 38 |
| Parity | | | | | | |
| 0 | 1.2 | (0.84, 1.6) | 67 | | | |
| 1 | 1.0 | Referent | 93 | 1.0 | Referent | 93 |
| 2 | 1.0 | (0.76, 1.3) | 145 | 1.0 | (0.79, 1.4) | 145 |
| 3+ | 0.91 | (0.69, 1.2) | 175 | 0.98 | (0.73, 1.3) | 175 |
| Age at first birth[a] | | | | | | |
| <20 | | | | 1.0 | Referent | 153 |
| 21-23 | | | | 1.3 | (1.0, 1.6) | 140 |
| >24 | | | | 1.3 | (1.0, 1.8) | 120 |
| Social status | | | | | | |
| low | 1.0 | Referent | 442 | 1.0 | Referent | 386 |
| high | 1.1 | (0.80, 1.6) | 38 | 0.91 | (0.60, 1.4) | 27 |
| Marital status | | | | | | |
| Never married | 1.2 | (0.85, 1.6) | 84 | 1.2 | (0.82, 1.7) | 46 |
| Married | 1.0 | Referent | 309 | 1.0 | Referent | 289 |
| Divorced/separated | 1.5 | (1.2, 1.9) | 82 | 1.6 | (1.2, 2.1) | 73 |
| Widowed | 2.0 | (0.80, 4.9) | 5 | 2.1 | (0.84, 5.2) | 5 |

[a] Not included in the model with all women. *n*, number of deaths.

the married. The other variables had effects consistent with the literature (van der Graaf *et al.*, 1988; Berrino *et al.*, 1995; Carmichael *et al.*, 1986). There was no difference in excess mortality between the various calendar periods, and no clear age effect emerged.

Few studies have investigated possible relationships between reproductive history and prognosis in cervical cancer patients. DeBritton *et al.* (1993) have reported on parity as a prognostic factor in women with cervical cancer in a Panamanian cohort study. In contrast to our results, these investigators found women with six or more pregnancies to have a 2.5-fold excess risk of dying compared with those with three or fewer pregnancies.

A Chinese ecological analysis reported a significant negative correlation between age at first birth and cervical cancer mortality, which reflects both the incidence and the survival rate (Guo *et al.*, 1994). No association, however, was found with number of live births. A British study on mortality in relation to child-bearing history found an increasing trend with increasing parity (Green *et al.*, 1988).

The good prognosis for mothers with an early first birth, as shown in the present data set was apparently not due to socioeconomic resources and family situation factors that have been thought to influence prognosis through access to medical treatment and care, and various sociodemographic factors (Vågerö and Persson, 1987; Goodwin *et al.*, 1987; House *et al.*, 1988; Ross *et al.*, 1990). Social and marital status were controlled for in the analysis. Early motherhood is also associated with low education and divorce, which would more likely contribute to a poor prognosis.

In the present analysis, in which age was included as a control variable, mothers with a low age at first birth would tend to have an older first-born child at the time of diagnosis. This might be a social or emotional advantage during treatment compared with having an infant or young child requiring close, and often quite exhausting, supervision and care. However, if this was an important factor, one should expect to find a stronger effect of the age of the youngest child at the time of diagnosis than of the age of the first born for women with at least two children. Separate models (not shown) were estimated for this subgroup, and again showed a significant protective effect of low age for the mother at first birth, but an adverse effect of high age for the child most recently born.

Another possible explanation was that women with an early first birth, given age and current parity, have had a longer interval between births. However, we could not discern any effect of an interval variable (not shown).

In summary, this study showed that parity level had no impact on the prognosis. However, the data suggested that having an early first birth might give a good prognosis for cervical cancer diagnosed many years after the delivery. This finding might be due to chance, or might be related to certain hormonal, nutritional and immunological changes imposed on the body during a pregnancy at an early age. Data from other studies on the relationship between reproductive factors and the prognosis in cervical cancer patients are sparse. Consequently, these relations should be further explored in other data sets.

**Acknowledgement**
This work was supported by Grant No. 95034/001 from the Norwegian Cancer Society.

### References

BERRINO F, SANT M, VERDECCHIA R, CAPOCACCIA R, HAKULINEN T AND ESTEVE J. (1995). *Survival of Cancer Patients in Europe. The EUROCARE Study*. International Agency for Research on Cancer: Lyon.

BJØRGE T AND KRAVDAL Ø. (1996). Reproductive variables and risk of uterine cervical cancer in Norwegian registry data. *Cancer Causes Control*, **7**, 351–357.

BJØRGE T, THORESEN SØ AND SKARE GB. (1993). Incidence, survival and mortality in cervical cancer in Norway, 1956-1990. *Eur. J. Cancer*, **29A**, 2291–2297.

BOSCH FX, MUÑOZ N, DE SANJOSE S, IZARZUGAZA I, GILI M, VILADIU P, TORMO MJ, MOREO P, ASCUNCE N, GONZALEZ LC, TAFUR L, KALDOR JM, GUERRERO E, ARISTIZABAL N, SANTAMARIA M, ALONSO DE RUIZ P AND SHAH K. (1992). Risk factors for cervical cancer in Colombia and Spain. *Int. J. Cancer*, **52**, 750–758.

BRINTON LA, REEVES WC, BRENES MM, HERRERO R, DE BRITTON RC, GAITAN E, TENORIO F, GARCIA M AND RAWLS WE. (1989). Parity as a risk factor for cervical cancer. *Am. J. Epidemiol.*, **130**, 486–496.

CARMICHAEL JA, CLARKE DH, MOHER D, OHLKE ID AND KARCHMAR EJ. (1986). Cervical carcinoma in women aged 34 and younger. *Am. J. Obstet. Gynecol.*, **154**, 264–269.

CLARK MA, NAAHAS W, MARKERT RJ AND DODSON MG. (1991). Cervical cancer: women aged 35 and younger compared to women aged 36 and older. *Am. J. Clin. Oncol.*, **14**, 352–356.

DEBRITTON RC, HILDESHEIM A, DE LAO SL, BRINTON LA, SATHYA P AND REEVES WC. (1993). Human papillomaviruses and other influences on survival from cervical cancer in Panama. *Obstet. Gynecol.*, **81**, 19–24.

GOODWIN JS, HUNT WC, KEY CR AND SAMET JM. (1987). The effect of marital status on stage, treatment, and survival of cancer patients. *JAMA*, **258**, 3125–3130.

GREEN A, BERAL V AND MOSER K. (1988). Mortality in women in relation to their childbearing history. *BMJ*, **297**, 391–395.

GUO WD, HSING AW, LI JY, CHEN JS, CHOW WH AND BLOT WJ. (1994). Correlation of cervical cancer mortality with reproductive and dietary factors, and serum markers in China. *Int. J. Epidemiol.*, **23**, 1127–1132.

HOPKINS MP AND MORLEY GW. (1991). A comparison of adenocarcinoma and squamous cell carcinoma of the cervix. *Obstet. Gynecol.*, **77**, 912–917.

HOPKINS MP AND MORLEY GW. (1992). The prognosis and management of cervical cancer associated with pregnancy. *Obstet. Gynecol.*, **80**, 9–13.

HOUSE JS, LANDIS KR AND UMBERSON D. (1988). Social relationships and health. *Science*, **241**, 540–545.

INTERNATIONAL FEDERATION OF GYNAECOLOGY AND OBSTETRICS. (1965). Classification and staging of malignant tumours in the female pelvis. *J. Int. Fed. Gynecol.*, **3**, 206–207.

INTERNATIONAL FEDERATION OF GYNECOLOGY AND OBSTETRICS. (1988). *Annual Report on the Results of Treatment in Gynecological Cancer*. Panorama Press: Stockholm.

KLEINE W, RAU K, SCHWOEORER D AND PFEIDERER A. (1989). Prognosis of the adenocarcinoma of the cervix uteri: a comparative study. *Gynecol. Oncol.*, **35**, 145–149.

KOSARY CL. (1994). FIGO stage, histology, histologic grade, age and race as prognostic factors in determining survival for cancers of the female gynecological system: an analysis of 1973–87 SEER cases of cancers of the endometrium, cervix, ovary, vulva, and vagina. *Semin. Surg. Oncol.*, **10**, 31–46.

KRAVDAL Ø AND HANSEN S. (1996). The importance of childbearing for Hodgkin's disease: new evidence from incidence and mortality models. *Int. J. Epidemiol.*, (in press).

KVÅLE G, HEUCH I AND NILSSEN S. (1988). Reproductive factors and risk of cervical cancer by cell type. A prospective study. *Br. J. Cancer*, **58**, 820–824.

LAMONT DW, SYMONDS RP, BRODIE MM, NWABINELI NJ AND GILLIS CR. (1993). Age, socio-economic status and survival from cancer of cervix in the West of Scotland 1980–87. *Br. J. Cancer*, **67**, 351–357.

LÄÄRÄ E, DAY NE AND HAKAMA M. (1987). Trends in mortality from cervical cancer in the Nordic countries: association with organised screening programmes. *Lancet*, **1**, 1247–1249.

MURPHY M, GOLDBLATT P, THORNTON JONES H AND SILCOCKS P. (1990). Survival among women with cancer of the uterine cervix: influence of marital status and social class. *J. Epidemiol. Community Health*, **44**, 293–296.

PARAZZINI F, LA VECCHIA C, NEGRI E, CECCHETTI G AND FEDELE L. (1989). Reproductive factors and the risk of invasive and intraepithelial cervical neoplasia. *Br. J. Cancer*, **59**, 805–809.

PISANI P, PARKIN DM AND FERLAY J. (1993). Estimates of the worldwide mortality from eighteen major cancers in 1985. Implications for prevention and projections of future burden. *Int. J. Cancer*, **55**, 891–903.

PRESTON D, LUBIN J, PIERCE D AND MC CONNEY M. (1993). *EPICURE-Risk Regression and Data Analysis Software Manual.* Hirosoft International: Seattle.

ROSS C, MIROWSKY J AND GOLDSTEEN K. (1990). The impact of the family on health: the decade in review. *J. Marriage Fam.*, **52**, 1059–1078.

STANHOPE C, SMITH J, WHARTON J, RUTLEDGE F, FLETCHER G AND GALLAGER H. (1980). Carcinoma of the cervix: the effect of age on survival. *Gynecol. Oncol.*, **10**, 188–193.

VAN DER GRAAF Y, PEER PG, ZIELHUIS GA AND VOOIJS PG. (1988). Cervical cancer survival in Nijmegen region, The Netherlands, 1970–1985. *Gynecol. Oncol.*, **30**, 51–56.

VÅGERÖ D AND PERSSON G. (1987). Cancer survival and social class in Sweden. *J. Epidemiol. Community Health*, **41**, 204–209.

ZEMLICKIS D, LISHNER M, DEGENDORFER P, PANZARELLA T, SUTCLIFFE SB AND KOREN G. (1991). Maternal and fetal outcome after invasive cervical cancer in pregnancy. *J. Clin. Oncol.*, **9**, 1956–1961.